# Moving Health Upstream in Urban Development: Reflections on the Operationalization of a Transdisciplinary Case Study

**DOI:** 10.1002/gch2.201700103

**Published:** 2018-08-07

**Authors:** Daniel Black, Gabriel Scally, Judy Orme, Alistair Hunt, Paul Pilkington, Roderick Lawrence, Kristie Ebi

**Affiliations:** ^1^ UWE Bristol Coldharbour Lane Bristol BS16 1QY UK; ^2^ Department of Economics University of Bath Claverton Down, Bath BA2 7AY UK; ^3^ Geneva School of Social Sciences Institute of Environmental Sciences University of Geneva 66 Boulevard Carl‐Vogt 1204 Geneva Switzerland; ^4^ Department of Global Health School of Public Health University of Washington Seattle WA 98195 USA

**Keywords:** impact, planetary health, transdisciplinary, upstream, urbanization

## Abstract

This paper describes the development, conceptualization, and implementation of a transdisciplinary research pilot, the aim of which is to understand how human and planetary health could become a priority for those who control the urban development process. Key challenges include a significant dislocation between academia and the real world, alongside systemic failures in valuation and assessment mechanisms. The National Institutes of Health four‐phase model of transdisciplinary team‐based research is drawn on and adapted to reflect on what has worked well and what has not operationally. Results underscore the need for experienced academics open to new collaborations and ways of working; clarity of leadership without compromising exploration; clarification of the poorly understood “impacts interface” and navigation toward effective real world impact; acknowledgement of the additional time and resource required for transdisciplinary research and “nonacademic” researchers. Having practitioner‐researchers as part of the research leadership team requires rigourous reflective practice and effective management, but it can also ensure breadth in transdisciplinary outlook as well as constant course correction toward real‐world impact. It is important for the research community to understand better the opportunities and limitations provided by knowledge intermediaries in terms of function, specialism, and experience.

## 1 Introduction

This paper describes a novel and informative transdisciplinary research collaboration that explores complex challenges in urbanization involving multiple, disparate disciplines, and sectors: linking quality of urban habitat to human health and associated economic cost, then exploring where and how health features—and might feature—in upstream decision‐making. Based on the Wellcome Trust pilot, “Moving health upstream in urban development” (“UPSTREAM”), we explore: project aims, objectives, and team; backgound rationale; project methods and outputs so far; key phases of transdisciplinary (TD) research and impact; observations on project development and conceptualization; and observations on the joint implementation of TD projects. We finish with a summary discussion and a list of recommendations.

## 2 Project Aims, Objectives, and Team

The aim of this pilot is to understand how human and planetary health could become a priority for those in control of urban development processes. The research proposal was developed to include: economic valuation to quantify the costs and benefits of good or poor quality urban environments using the latest economic valuation to provide decision‐makers with more comprehensive inputs of cost‐benefit analyses; real world engagement with those in control of the development process to understand the barriers and opportunities in creating healthy human habitats (led by experienced real world practitioners in partnership with experienced research colleagues).

The stated objectives are:

- To demonstrate to decision‐makers the hidden costs to society of poor quality urban development, using a monetary metric that allows comparison between the total health costs associated with exemplar and standard urban development.
- To test what impact the monetary value of health outcomes may have on the actions of a range of different public and private landowners and developers.
- To identify the barriers—and test potential opportunities—facing those landowners and developers who attempt to integrate health benefits in their decision making.
- To validate this taxonomy of barriers and opportunities through use of expert advisory groups with experience of urban development in the UK and overseas.
- To endorse the resulting strategy for the integration of health into urban development planning, with a particular focus on those in control of development.
- To disseminate the results nationally and internationally, targeted particularly those who have most influence over strategic development decisions.

The pilot focuses on the UK context only, but as a next step will seek to validate the findings in an international context.

**Figure 1:**
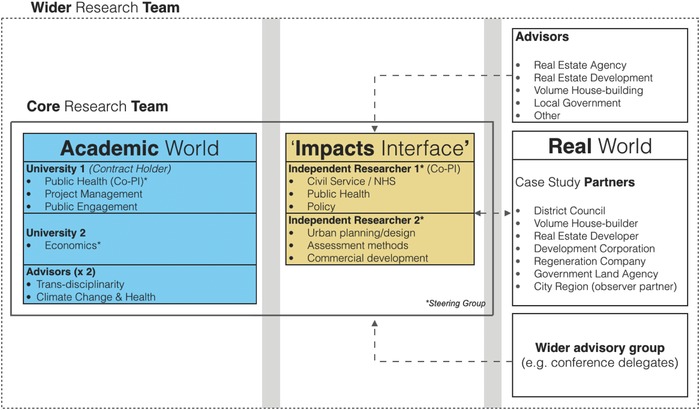
Core and wider research teams, institutions, competencies, steering group and the “impact interface.”

The research is impact‐focused and the team is co‐coordinated (and the various workstreams integrated) by a steering group that includes experienced university academics in public health and economics as well as independent researchers with significant real world experience in public health, urban development, and corporate decision‐making. The research team includes experts in air quality, energy in the built environment, health impacts of climate change, science communication, and transdisciplinarity. **Figure** **1** sets out the core and wider teams, their institutions, core competencies, their real world partners and the “impacts interface.”

With regards key aspects of terminology, we assume interdisciplinary also encompasses transdisciplinary working practice, and we are primarily interested in real world “external” impact, rather than academic impact (more specifically, we are focused on external impact in relation to human and planetary health).^1^, ^2^, ^3^, ^4^, ^5^ In addition, there is no accepted definition of the use of the term “upstream” when referring to the impact of the built environment on health. Frumkin uses the term “upstreamism” to describe the urban environment (in relation to health impacts downstream), while our pilot study and this paper uses the term “upstream” to define the agents, decisions, and processes even further upstream that determine the quality of that urban environment, which we then define as “midstream.”^6^, ^7^

## 3 Background Rationale

The relationship between health and the built environment has a long and well known history, particularly relating to infectious diseases, yet the link to emerging noncomuncable diseases are only partially understood, and the potential solutions to these issues often appear intractable (or “wicked”).^1^, ^2^, ^3^, ^6^, ^8^ Research in this area does not yield clear results from methods relying solely on linear causality, and there is widespread consensus for the need for whole‐systems analyses, and that effective solutions are more likely to be found by addressing the urban environment rather than narrowly focusing on healthcare.^9^, ^10^, ^11^, ^12^, ^13^, ^14^, ^15^, ^16^, ^17^, ^18^, ^19^, ^20^

Methods used to improve quality of urban environments—such as design guides, and assessment methods—are relatively impotent against and peripheral to the fundamental drivers of urban development.^21^, ^22^, ^23^ The reasons for this are highly complex. Globalization and laissez‐faire governance provide a context in which urban development is driven largely by global resource flows of capital, ownership, and personnel.^24^, ^25^ Economic valuation is an important part of decision‐making, yet it ignores factors crtical to human and planetary health; Stern described climate change as “the greatest market failure ever seen,” a view echoed by Mark Carney, the Governor of the Bank of England, in 2015.^26^, ^27^, ^28^, ^29^, ^30^, ^31^ Global challenges such as these are leading some to question whether current models of corporate governance are outdated and need revising.^32^, ^33^

The research domain, and especially its discipline‐based protocols and methods, is currently not adequately configured to deal with these challenges. Crossing boundaries horizontally (specialisms), vertically (tiers of decision‐making), and between worlds (academia and the real world) requires a significant change in research practice. Some have proposed more inter‐ and transdisciplinary research, as well as new methods of inquiry, funding and research impact assessment including both monetary and nonmonetary values.^34^, ^35^ Interdisciplinary projects that explore this nebulous “impacts interface” are needed to navigate effective pathways to impact; this has yet to be clearly mapped.^5^, ^35^, ^36^, ^37^ Collaboration among investigators from different disciplines and fields is challenging enough, but can be even more dramatic when a team includes both investigators and translational partners who often have divergent opinions and expectations about the goals of the translational partnership, each other's status as team members, and each group's potential contributions to the team's activities.^36^ Knowledge intermediaries are often endorsed uncritically, and there is “no knowledge broker certificate,” but they and their knowledge can be, and often are, critical to achieving external real world impact from research.^36^, ^37^, ^38^, ^39^, ^40^, ^41^, ^42^, ^43^

**Figure 2:**
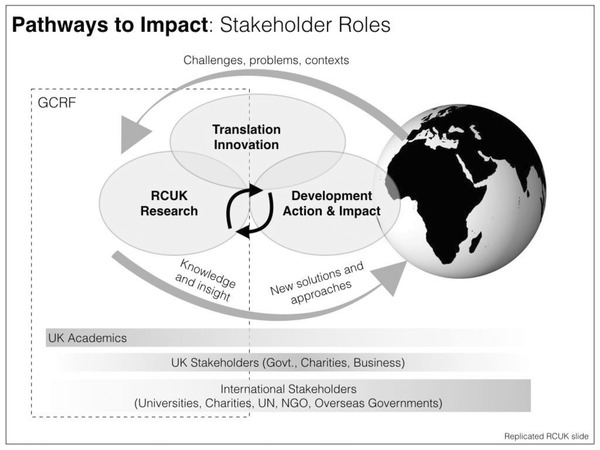
RCUK segregation of funding, stakeholder roles, and conceptual pathways to impact.

**Figure 3:**
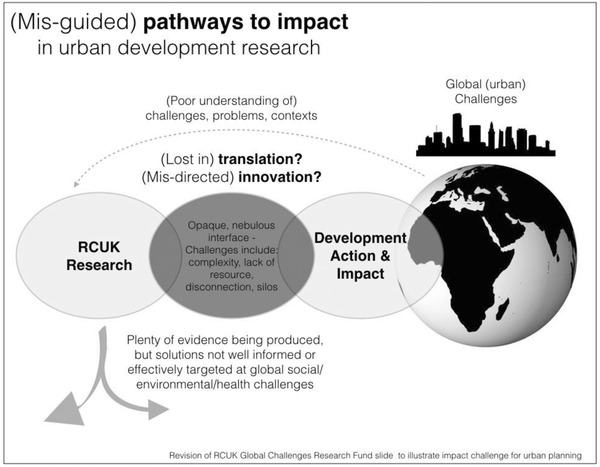
Comparative illustration of RCUK Pathways to Impact showing disconnection between academia and real world in achieving real world impact in urban development.

The recent and increasing focus of funding agencies on external impact highlights the dislocation of academia from the real world and the limited effect much current research has been having on human and planetary health.^37^, ^44^ In urban development, the dislocation is pronounced. We use **Figures** **2** and **3** to illustrate the issues facing academia and research funders in mapping effective pathways to impact in urban development: in reality, the complexity of the challenges, problems, and contexts are not comprehensively understood by academia, nor is the translation/innovation space clearly defined or well understood.^5^, ^6^, ^9^, ^11^, ^16^, ^18^, ^22^, ^24^, ^25^, ^30^, ^31^, ^32^, ^33^, ^37^, ^38^, ^39^, ^40^, ^41^, ^42^, ^43^

While urban living is enjoyed by many, particularly in the more affluent urban areas, truly healthy urban environments are extremely rare.^3^, ^12^, ^17^, ^19^, ^45^ Reasons for this are also highly complex: aspirations are “frustrated by outdated public policies and conservative fund‐holders”; a common theme in better quality development is that the primary controlling agency, usually the landowner, is “committed to the objective of creating a better form of urbanism.”^30^, ^31^, ^32^, ^33^, ^46^, ^47^ One global exemplar has developed on public land in Vauban district of Freiburg, Germany's fastest growing city, where among other exceptional outcomes rate of car use is 16% compared to 32% in Freiburg as a whole and a national average in Germany of 85%.^48^, ^49^, ^50^, ^51^ Critical success factors include strong links between landowner and community, active community involvement and leading technical expertise.^48^

A very significant challenge facing those seeking to enable healthy urbanization is the timescales involved, not least in evidencing impact. Even relatively small neighborhoods take decades to build and many decades more for the health outcomes to be felt. We need to move beyond the “discovery myth” and develop new methods of (current and future) impact assessment.^5^, ^52^, ^53^

This emerging evidence, from academia and the real world, suggests a system of urban development delivery and governance that is having a significant negative impact on human and planetary health without considering it a priority. Systemic challenges such as these require examination not just of mid‐stream factors such as the design of the built form itself, but of the upstream agencies that control the quality of that environment.

## 4 Project Methods and Outputs

The project has two main design strategies: the economic valuation component, which is quantitative and nonexperimental; the exploration of barriers to, and opportunities for, change (and the development of an endorsed strategy) that is qualitative, experimental and “action‐research” based. The large scale of neighborhood‐level development projects and the relative inaccessibility of strategic level decision‐makers justifies a case study approach using a purposive sample of real world urban development delivery agencies.^54^

The economic valuation is based on available evidence linking quality of built form to health outcomes. To that end, and as a first step, a comprehensive literature review was undertaken by public health specialists that searched under five separate thematic areas—transport, buildings, neighborhood design, natural environment and food—across eight electronic databases: MEDLINE, PsychINFO, Cumulative Index to Nursing & Allied Health Literature, Applied Social Sciences Index and Abstracts, Cochrane Database of Systematic Reviews, SocINDEX, Econlit, and Allied and Complementary Medicine. Each thematic area revealed subthemes (e.g., evidence on neighborhood design linked to issues of walkability, accessibilty, connectivity, and amenity) and the strength of the evidence was determined in each area using the Effective Public Health Practice Project tool, which consists of six domains for quality assessment: 1) probability that the study participants are representative of the target group (selection bias); 2) design of the study; 3) control of confounding factor; 4) concealment of participants and researchers (blinding); 5) reliability and validity of data collection methods; 6) reporting of withdrawals and dropout rate.^55^ Of the 22 428 papers originally found, 209 met the search criteria. Evaluating the literature sets out the quality and nature of published evidence; it will enable a comprehensive evidence gap analysis; it forms a core part of our economic valuation; and it adds detail and nuance to the existing knowledge base. **Table** **1** below provides a summary overview of findings linking urban form to health outcomes and the strength of evidence in each case.

**Table 1:** Table showing headline findings from literature review and strength of evidence

Categories	Key themes	Outcomes	Strength of evidence (S/M/W)
Neighborhood Design	Increase neighborhood walkability	Reduced risk of hypertension (S)	S
		Reduced risk of diabetes and prediabetes (M)	M
		Improved mental health (M*)	M
	Increase access to facilities and amenities	Increased physical activity levels (M),	M
		Improved mental health (S)	S
	Enhance neighborhood connectivity	Reduced limitations in performing instrumental activities of daily living among men (M)	M
		Reduced risk of obesity among women (W)	W
	Improve access to open green space	Improved mental health (S)	S
		Reduced risk of non accidental mortality (S)	S
		Increased physical activity levels (S*)	S
		Reduced cardiovascular risk factors (S)	S
		Reduced risk of asthma (M*),	M
		Reduced risk of diabetes and prediabetes (M)	M
Buildings	Improve thermal quality and ventilation	Improved general health and respiratory outcomes (S*)	S
		Reduced blood pressure (S*)	S
		Reduced cost associated with heating(S)	S
		Reduced level of NO_2_ in the living room (M)	M
		Reduced mold contamination (M)	M
		Improved school attendance among children (M)	M
	Improve quality of housing (health and safety)	Reduced falls and fall related injuries among older adults (S)	S
		Improved mental health (M)	M
	Inadequate quality of housing	Increased mortality from coronary heart diseases (M)	M
	Increase access/relocation to affordable homes or social housing	Improved general health among previously homeless people (M)	M
		Improved mental health among adults and children (M)	M
		Improved educational achievement among young boys (M)	M
Natural environment	Exposure to environmental hazards (air pollutants)	Increased risk of cervical cancer (S*)	S
		Increased risk of brain cancer (S)	S
		Increased risk of non‐accidental mortality (S*)	S
		Increased risk of lung cancer (S)	S
		Increased blood pressure (M*)	M
		Increased risk of dementia and Alzheimer's disease (M*)	M
		Increased risk of type II diabetes (M)	M
	Reduce exposure traffic noise	Increased average life expectancy (M)	M
		Economic savings of 9.3 billion EUR per year (M)	M
	Improve access to green space	Improved respiratory outcomes (S*)	S
		Increased physical activity level (W)	W
		Improved mental health (W)	W
	Exposure to traffic noise	Increased risk of myocardial infarction among males (S)	S
		Poor academic performance among children (S)	S
		Reduced quality of life among women (S)	S
		Increased blood pressure (S)	S
		Worsened mental health (M)	M
		Increased risk of Type II diabetes (S)	S
Transport	Improve infrastructure for walking and cycling	Increased physical activity levels (S*)	S
		Reduced risk of pedestrian motor vehicle collision (S*)	S
	Improve road safety	Reduced risk of pedestrian injury (S*)	S
		Reduced risk of road traffic collision (S*)	S
	Improve public transport Infrastructure	Increased active transport (M)	M
		Reduced exposure to road traffic collision (M)	M
	Exposure to traffic related environmental hazards	Increased risk of pre/postmenopausal breast cancer (S)	S
Food	Increase access to healthy food environment	Reduced odds of obesity (M*)	S
	Density and proximity of fast‐food outlets	Increased risk of diabetes (S*)	S
		Increased risk of obesity (M*)	S
	Exposure to unhealthy food outlets near school environment	Increased risk of obesity (W)	S

The publications derived by the literature review were then examined by the economists for usable data to populate their valuation, and relinked to new thematic areas based on available evidence of costs associated with the health outcomes identified. **Table** **2** shows the number of studies related to each new economic subtheme, and it also reveals gaps in evidence. For example, while there is a good deal of evidence (that can be valued economically) on air pollution (67 related studies), there is very little on overheating (3 related studies). The development of the economic valuation methodology is still in process. It draws on this health evidence and is being designed to support real world decision‐making processes.

**Table 2:** Table showing number of studies derived through the literature review that can be used by the economic valuation broken down in to revised subthemes

Typology characteristics	No. studies
Air quality	67
Walkability	21
Noise	18
Green space—amount (inc. access and proximity)	17
Road safety	16
Housing affordability	11
Cycling	10
Damp	9
Fast food outlets	9
Cold	8
Supermarket	7
Fear of serious crime	7
Green space—quality	7
Amenities within walking distance (walk score)	6
Ventilation	6
Convenience stores/small shops	5
% socioeconomic status of area	5
Public transport links	5
Proximity to main road	5
Overheating	3
Recreational space/playgrounds	2
Sports provision	2
Falls intervention	2
New or regeneration	2
Renewal of interiors	2

The final phase of the research focuses on two rounds of interviews with senior executives, decision‐makers, and other key knowledge holders. Our case study group represent most of the UK's major urban delivery agencies: volume‐house builder; regeneration company; city council; city region; district council (responsible for one of the NHS's “Healthy New Towns”); development corporation; real estate developer.

## 5 Key Phases of Transdisciplinary Research and Impact

**Figure 4:**
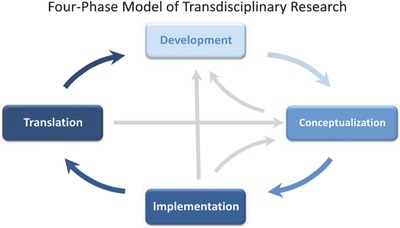
Hall et al. four‐phase model of transdisciplinary team‐based research.

In order to describe the successes and challenges relating to the operationalization of this research pilot, we begin by presenting the broad stages of the research process. Juxtaposing the evolution of our emerging model against another previously described allows us to illuminate distinguishing features. While there is no standard research process formula, there tend to be types of activity common to many, and in the inter/transdisciplinary literature various models have been proposed.^34^, ^36^, ^56^, ^57^ We draw on the “four‐phase model of transdisciplinary team‐based research,” derived from the evolving “Science of Team Science” (SciTS) programme of work, as it is the most comprehensive, accessible and suited to our purposes of those reviewed (**Figure** **4** and **Table** **3**).^58^, ^59^ Developing from within the health sector, the emerging SciTS field draws heavily on team research from other fields including business, military, and sports, and recognizes additional value from cross‐disciplinary working in fields as diverse as climate change to genomics. SciTS seeks to: a) measure the outcomes of team research and b) guide scientists in building effective teams, including consideration of issues relating to suitability of team or individual, size of team, resource, training and authorship.^36^

**Table 3:** Hall et al four‐phase model of transdisciplinary team‐based research with goals, critical components, team type, and key team processes

	Developmental	Conceptual	Implementation	Translational
Primary goal	Establish a shared understanding of the scientific or societal problem space of interest—including what concepts fall inside and outside its boundaries—and mission of the group	Develop novel research questions or hypotheses, a conceptual framework, and a research design that integrate and extend approaches from multiple disciplines and fields	Launch, conduct, and refine the planned TD research	Apply research findings to advance progress toward developing innovative solutions to real‐world problems, as appropriate to the level of science at which the research is conducted
Team type(s)	Network	Emerging team	Real team	Adapted team
	Working group	Evolving team		New team
	Advisory group			
	Emerging team			
Key team processes	Generate a shared mission and goals	Create a shared mental model	Develop compositional, taskwork, and teamwork transactive memory	Adapt the team, as needed, to address translational opportunities
	Develop critical awareness	Generate shared language	Conflict management	Generate shared goals for the translational endeavor
	Externalize group cognition	Develop compilational transactive memory	Team learning	Develop shared understandings of how these goals will be pursued
	Develop a group environment of psychological safety	Develop a team TD orientation		

**Figure 5:**
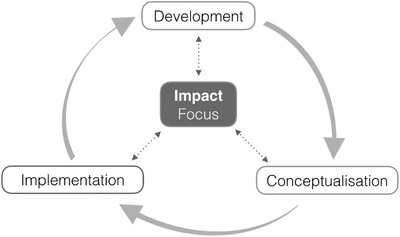
A revised three‐phase impact‐focused model, which does not have “translation” as a fourth phase, but rather impact has a constant and central focus within the evolving method development.

We have adapted this four‐phase model by removing the translation phase, which is not a separate phase, but a critical focus throughout (**Figure** **5**). We move beyond basic research to “problem‐focused research”—which is defined as “distinct from what is called “free” or “basic” research because it is “field‐induced”…(it) emphasizes usefulness, efficiency, and practical results.”^57^

## 6 Development and Conceptualization

The four‐phase model sets out the following key overlapping activities in the developmental and operational phases: 1) generating a shared mission and goals (then creating a shared mental model); 2) developing critical awareness (then developing a team TD orientation and shared language); 3) externalizing group cognition (then development of a “compilational transactive memory”); and 4) developing a group environment of psychological safety. A key distinguishing feature between the initial development phase and the conceptualization phase was, in our case, the development of the original vision. We combine the first two phases here because the key activities described overlap considerably in terms of reflecting on the operationalization of the research.

### 6.1 Generating a Shared Mission and Goals

The developmental stage is often initiated by an “individual or small core group motivated to advance the science and/or practice in a particular area area.” In this pilot, a critical difference was that these “originators” were not career academics fixed within an institution, nor do they fit comfortably in to what Hall et al. describe as “stage‐4 translation,” which implies communication and lacks definition on issues such as sector‐specific expertise, level of real world experiene or research leadership.^5^, ^36^, ^40^ The experience, capacities, and responsibilites of these “nonacademic” research leads has been of fundamental importance in this study. Though knowledge brokerage is now a relatively well‐established area of activity, particularly in health research, we have observed significant gaps in understanding amongst researchers and funders with regards real world impact in urbanization.^37^, ^38^, ^39^, ^40^, ^41^, ^42^

The goal of the project, based on a shared understanding, was that a fundamental blockage in creating healthy urban environments lay not “mid‐stream” with the professional classes and the design of the built form itself, but with the power centres and agencies further “upstream” who exercised most control over the development and management of the urban environment.^21^, ^22^, ^23^, ^24^, ^25^, ^26^, ^27^, ^28^, ^29^, ^30^, ^31^, ^32^, ^33^

### 6.2 Developing Critical Awareness

Much has been written on increasing researchers' capacity to enable more effective transdisciplinary (TD) working practice, including what is referred to as “TD intellectual orientiation,” which relates to core attitudes, beliefs, values, cognitive skills, and behaviors. Critical awareness—the “understanding that all disciplines and fields have substantive and methodological strengths and limitations”—and developing a team TD orientation was inherent to our original team principles and, to date at least, has not needed to be voiced in great detail.^36^ On reflection, this may be due to a combination of four factors: first, the relatively diverse background of the real world originators who naturally draw (and rely) on specialist expertise from a range of disciplines; second, the funding requirement to explicitly embrace transdisciplinarity, and the recruitment to the team of a specialist in transdisciplinary research; third, the openness to transdiscplinary working of the original team; and fourth, the incubation of the project in an institution that values its expertise in applied research and real world interaction. Developing a shared understanding and language has been a small challenge however, and this has surfaced more in the implementation phase, which we cover below.

### 6.3 Externalizing Group Cognition

With regards to the externalization of group cognition and development of an (opaquely titled) “compilational transactive memory,” we largely achieved this but, if undertaken more comprehensively, it could have been a valuable tool in the recruitment process. Hall et al. discuss using structured approaches such as systems dynamics modelling and concept mapping to enable group members to develop a “cognitive artifact” and to “clearly identify the scope of the problem space as well as the relevance of each member's expertise…while working toward consensus about the overarching boundaries of the potential collaborative endeavour.”^36^ As described above, due to shared real world experience and values, identification of the problem space was also not a challenge for the Steering Group.

We would however support the use of a more rigorous mapping of the problem space to team expertise, as it might counter‐balance the tendency, common to all large institutions—whether academic, private, public or third sector—toward maximization of internal staff capacity and satisfaction of target‐driven cost centres, as opposed to the distribution of resources to the most appropriate people, whether inside or outside the institution. There are, of course, efficiencies of scale when working within large institutions: large organizations can offer a full range of specialist service personnel experienced in working together in‐house, which can save time, money and, hopefully in many instances, provide a better service. Furthermore, there are inefficiencies in pulling together diverse new teams each time who have to learn as they go. Arguably however, those efficiencies of scale are maximized in well‐trodden areas of activity. In the case of exploratory transdisciplinary research, there are greater gains to be made by allowing and encouraging that diversity of grouping.^8^, ^9^, ^10^, ^11^, ^23^, ^34^, ^35^, ^36^, ^37^, ^38^, ^39^, ^40^, ^41^, ^42^, ^43^, ^44^, ^54^, ^56^, ^57^, ^58^, ^59^, ^60^, ^61^

It is perhaps worth reiterating therefore that, despite the inevitable bureaucratic requirements, the diversity of grouping in our pilot was made possible by flexible approaches to the incubation of transdisciplinary working with independent researchers external to the university. Even after many decades of inter‐ and transdisciplinary research, “conceptual and institutional barriers for transdisciplinary inquiry are still common (and) incentives remain rare…not only due to the scepticism of decision makers in academic institutions, in conventional funding agencies and in policy decision making, but also to the formal education and personal motives of scientific researchers in academic institutions.”^62^

### 6.4 Developing a Group Environment of Psychological Safety

The concept of “psychological safety” is pertinent, particularly in this era of increasing mental health problems such as anxiety and depression, and also because some team members worked in the increasingly pressurized work environments of higher education instititions. This is not something we considered in the early developmental phase, but we consider this in the formulation of the implementation phase.

## 7 Implementation

The key activities from the four‐phase plan are: 1) develop compositional, taskwork, and teamwork transactive memory; 2) conflict management; and 3) team learning. All three of these activities merge in to one in consideration of this pilot. The implementation phase overlaps with the adjoining phases, but has been sufficiently distinct within this case study to be covered separately.

### 7.1 Developing Transactive Memory

The first two (quantitative) phases of our pilot—the literature review and the economic valuation—were using established methods and therefore were, in isolation, not requiring of substantial development. A key early challenge however (and the transdisciplinary nature of this part of the project) was in the merging of the two; a core objective of the pilot is to develop an economic valuation of the costs and benefits of health outcomes from low and high quality urban forms. In this case, it took time for “a shared understanding of who knows what, who does what” to be developed alongside an alignment of these two academic specialisms and their outputs towards real world relevance and impact.

An expected, but nonetheless surprising lesson learned from this project has been the very significant length of time (and associated resource) it takes to develop a merged approach, not only between disciplines, but also when integrating between academia and the real world. Though technology now allows relatively excellent communication across even large distances, and good levels of interaction are not always guaranteed solely because of geographical proximity, the level of interaction required for transdisciplinary research does require greater resources, not least when seeking to resolve new challenges, which may require high levels of interaction between multiple individuals.^36^ Although perhaps an obvious point, it is worth underlining that, in general, funding agencies do not allocate resources specifically for the additional time required for interpersonal communication during inter‐ and transdisciplinary projects in comparison to conventional discipline‐based projects.

Coordination of the activity between two work groups was important and required not only the generalist knowledge of the real world project coordinator, but also the academic expertise with regards to ensuring rigour of approach. It is unclear whether it might have been more efficient to engage in more comprehensive briefings of the different specialisms beforehand; an acceptance that these new collaborations take time is important, both for funder and researcher.

A key lesson taken from this process of merging distinct fields of expertise has been knowing not just when to involve team members, but when not to. When working across mutliple teams and disciplines, effective communication (which ensures that all partners are fully briefed) is not only very important, but also a constant challenge. Sometimes however, having too many ideas in one room can pull discussions away from the core direction of the method development and focused discussion between key team members is needed to resolve it; we found that by reducing the number of staff to the core team responsible we made significant progress again. Reflecting on the purpose of each meeting and who was best to involve is clearly important, although it may not always be possible to see this in advance and inclusivity should not be compromised because of a misjudged sense of what constitutes efficiency. The process should be a constant process of reflection and judgement.

### 7.2 Conflict Management

The gap between specialisms emerged again in the development of the (qualitative) interviewing method. Initially, there was disagreement as to the core nature of the approach, which was resolved when an approach was found that fitted with the specific project requirements. Determining this method in advance may have precluded this becoming an issue, but due to the evolutionary nature of the project, it is also possible that the appropriate method could only have revealed itself at the stage it did. For similar reasons, a later challenge has been in the development of a shared understanding of terminologies used for the coding of the interviews, which relates again back to the developmental and conceptualization phases, agreeing a shared vision and fitting the team to the problem space. A solution has been formulated through an agreed process that will enable iterative cross‐checking of shared meaning and understanding prior to analysis of findings. As with the selection of interview methodology, the coding mechanisms might have been determined in advance in more detail, but it is not clear at this stage how much time it would have saved, if any.

A temporarily significant issue was around lack of clarity over leadership (and authorship). Due to the unusual nature of the project and team set‐up, project leadership had been shared three‐ways: with the originators, who were outside the lead academic instititution, and across two departments within the university, one which was leading the academic work and another which held the administrative contract. Despite careful consideration of management hierarchy during the proposal, and the early establishment of comprehensive management processes, what had not been foreseen were the disagreements between leading parties over the strategic direction of the project and the appropriate methods to be used.

### 7.3 Team Learning

These conflicts and related debates used up time and resource, but have been resolved and have led to “new perspectives and new knowledge,” have ultimately been “helpful for making strategic decisions,” and have led to a stronger and “emergent team characteristic.”^36^

Given the significant level of interactions combined with the evolving nature of the method development, project management and administration have been arguably more important on this transdisciplinary pilot than perhaps is usual for a research project. Having a dedicated project management/administration function (probably separate from the research function) that reports directly to and is line managed by the project coordinator is important. If resource is available, this would free up the specialist researchers to get on with what they are most interested in, though this would be an additional subcontract cost where coordination is external to the research institution and may not align with the financial calculations of academic institutions.

## 8 Discussion

As set out early on this paper, the research team included those in the academia world, those in the “impacts interface” and those from the real world. Critical to the success of our research project has been the partnership between the “impact interfacers” and leading academics who have the confidence, experience and flexibility to think openly and creatively about the research agenda, to embrace complexity, and to ensure rigour through what has been a constantly shifting set of challenges and constraints.

Although we have yet to monitor external real world impact from the research work, the benefits of having experienced real world practitioners as part of the research team's steering group appears clear:, e.g., strong links to industry partners and advisors; constant sense‐checking of real world relevance and focus on impact; ability to coordinate across multiple disciplines; ability to communicate effectively with senior industry partners (in this case, to undertake interviews with senior executives). Miller et al. advocate greater integration between real world practiioners and Robson flags three benefits of the “practitioner‐researcher” role: “insider” opportunities, “practitioner” opportunities and “practitioner‐researcher” synergy.^63^, ^64^

We have perhaps been fortunate that the challenges we have faced on this pilot so far have been relatively minor, and we have given reasons why that might be the case.

However, while there are undoubted benefits of transdisciplinary (and transinstitutional) working practice, there are clearly improvements that can be made. In particular, we have discovered that, when engaging in transdisciplinary, transinstitutional team work, clarity around leadership and effective project management may need greater attention. That said, these minor hurdles have certainly not been insurmountable and simple solutions are available as long as institutional funding requirements permit.

With regards to ensuring successful conflict resolution, the concept of “psychological safety” flagged in the first—“developmental” phase of the four‐phase model, is to a certain extent pertinent to this case study, although not in the way the authors describe. *Psychological safety* is described by them as a “*belief that the team operates in an environment where members feel comfortable expressing independent thoughts and opinions as well as divergent assumptions about the nature of varied research approaches, without fear of embarrassment, rejection, or punishment. Psychologically safe team environments promote active listening and debate and discussions that are characterized by open sharing of ideas and mutual respect*.” None of this has been a challenge for us. Our team inherently fosters “*a working environment that enables group members to understand and acknowledge differences in their disciplinary perspectives and values, engage in colearning among disciplines, and move on to identify common ground for the collaboration*.” That said, the lack of clarity around leadership and project management have created some additional confusion and pressure, and in academia where mental health problems, particularly stress and anxiety, apears to be higher than the average, placing emphasis, both early and regularly in the process, on ‘psychological safety' would seem sensible.^65^

The four‐phase model we have drawn on in this paper has been extremely useful, not necessarily in drawing out these reflections, but certainly in framing them in a clear and coherent manner. Using such a model may have enabled a better planned undertaking, but conversely the parameters, albeit very useful, may also have constrained exploration. A useful avenue of study would be to determine how much the freedom to create new methods of transdisciplinary practice without being constrained by predetermined models (or funding requirements) might be a factor in the successful operationalization of research results that make a difference when applied in professional practice.

## 9 Recommendations

Key insights and suggestions from the project are as follows:

1. Early use of the four‐phase model could help research teams to plan more effectively, particularly in the development phase and with regards to team assembly.
2. Differentiating between upstream and midstream is important when considering complex urban systems of development and governance.
3. A better understanding of the opportunities and limitations provided by knowledge intermediaries in terms of function, specialism and experience could enable researchers, funders and assessors to fast track impact.
4. Partnering with experienced generalists can help specialist academics in developing their TD orientation and critical awareness.
5. Academic institutions that are open to working in new ways are more likely to sieze new opportunities in transdisciplinary research.
6. Rigourous reflective practice, effective management, and clear communications are all critical when exploring the impacts interface, and should help significantly in ensuring a shared understanding and language, and “psychological safety”; having graphical skills in the leadership team can help in that communication; knowing when to include team members and when not to is a constant challenge and requires clarity of thinking, time and experience.
7. Exploring new ways of working will tend to require expansive, outward‐reaching, transinstitutional working practice, which may require transcendence of existing institutional infrastructures and target‐driven requirements.
8. Experience and openness is required to ensure clarity of leadership without compromising ability to explore; regular steering group meetings can help significantly in resolving.
9. Recognition is needed, by both researchers and funders, of the potential benefits from and greater resource requirements in transdisciplinary research.
10. Shared leadership offers multiple benefits, but line management can become blurred, particularly when transinstitutional, but also when transdepartmental, so clear definition of roles and responsibilities early on with regular revision and reporting to Steering Group is important.

## Conflict of Interest

The authors declare no conflict of interest.
